# Comparison of validation protocols for blood pressure measuring devices in children and adolescents

**DOI:** 10.3389/fcvm.2022.1001878

**Published:** 2022-11-23

**Authors:** Stella Stabouli, Athanasia Chainoglou, Kleo Evripidou, Carla Simão, Christina Antza, Panagiotis Petrou, Gilad Hamdani, Javier Calpe, Empar Lurbe

**Affiliations:** ^1^1st Department of Pediatrics, Faculty of Health Sciences, School of Medicine, Hippokration General Hospital, Aristotle University of Thessaloniki, Thessaloniki, Greece; ^2^Department of Pediatrics, Faculty of Medicine, Hospital Universitario de Santa Maria, FMUL, Lisbon, Portugal; ^3^3rd Department of Medicine, Faculty of Health Sciences, School of Medicine, Papageorgiou Hospital, Aristotle University of Thessaloniki, Thessaloniki, Greece; ^4^Pharmacoepidemiology-Pharmacovigilance, Pharmacy School, School of Sciences and Engineering, University of Nicosia, Nicosia, Cyprus; ^5^Nephrology and Hypertension Institute, Schneider Children’s Medical Center, Petah Tikva, Israel; ^6^Analog Devices, SLU, Paterna, Valencia, Spain; ^7^Department of Pediatrics, Centro de Investigación Biomédica en Red (CIBER) Fisiopatologia Obesidad y Nutricion, Instituto de Salud Carlos III, University of Valencia, Valencia, Spain

**Keywords:** blood pressure, device, validation, children, adolescents

## Abstract

Accuracy of blood pressure (BP) measurement is important for the evaluation of hypertension in children and adolescents, and it is critically dependent upon the accuracy of the BP measuring device. A device that could pass validated protocols with reliable accuracy would be desirable in clinical and research settings. Several scientific organizations have published recommendations on the validation of different BP measuring devices. Most of them focus on adults but separate recommendations and validation criteria for BP devices intended for use in children and adolescents are included in some validation protocols. In this review, we compare the validation criteria for BP measuring devices among consensus documents from different scientific organizations focusing on the pediatric population and we discuss the evidence gaps targeting the needs for validated BP measuring devices in children and adolescents. We also highlight common pitfalls in the validation studies of BP measuring devices in children and adolescents using the example of office BP devices.

## Introduction

Accuracy of blood pressure (BP) measurement is important for the evaluation of hypertension in children and adolescents, and it is critically dependent upon the accuracy of the BP measuring device. The need for evaluation of the accuracy of automated BP measuring devices available in the market, both for use in clinical settings, as well as out-of-office environment, using validation procedures has been well-recognized by the scientific community and the manufacturers (1). A device that can pass validated protocols with reliable accuracy would be desirable in clinical and research settings.

Several scientific organizations have published consensus documents on the validation of BP measuring devices. First, in 1987, the American Association for the Advancement of Medical Instrumentation (AAMI) published a monograph on clinical validation procedures for automated BP monitors, which was recognized as a national standard in the United States (2). This protocol was subsequently revised in 1992 and 2002. In 1990, the British Hypertension Society (BHS) published another validation protocol for BP monitors, which was revised in 1993 (3, 4). The German Hypertension League (DHL) developed its own Quality Seal Protocol in 1999 (5), and in 2002, the European Society of Hypertension (ESH) introduced the ESH-International Protocol (ESH-IP) that was revised in 2010 (ESH-IP2) (6, 7). In 2009, the International Organization for Standardization (ISO) published its own protocol (8) and in 2013 the American National Standards Institute (ANSI), the AAMI, and the ISO collaboratively released a revised protocol (9). Finally, in 2018, the AAMI/ESH/ISO released the most recent validation protocol in an effort to develop a single universal protocol for the validation of BP monitors (10).

Most of the validation protocols are developed only for adults and children are regarded as a special population requiring separate validation studies. The main objective of these consensus statements was to provide practical guidance for validation studies of BP measuring devices and to ensure that conditions are fulfilled, and data are reported in detail. Still, despite previous and current recommendations performing and reporting on validation studies present significant limitations. The pitfalls are more pronounced when the validation studies are performed in children and adolescents (11).

In this review, we compared the validation criteria for BP measuring devices among the consensus documents from scientific organizations focusing on the pediatric population and we discuss the gaps in evidence targeting the needs for validated BP devices in children and adolescents. We also highlight common pitfalls in the validation studies of BP measuring devices in children and adolescents using the example of office BP devices.

## Differences between validation protocols

The basic differences between the validation protocols are summarized in Table 1 and include the following key features:

**TABLE 1:** Comparison of validation protocols for blood pressure (BP) measuring devices.

	BHS (3)	Quality seal protocol -DHL (4)	ESH IP2 (5)	ANSI/AAMI/ISO (7)	AAMI/ESH/ISO (8)
Organization	British Hypertension Society	German Hypertension League	European Society of Hypertension	American National Standards Institute/Association for the Advancement of Medical Instrumentation/ International Organization for Standardization Collaboration	Association for the Advancement of Medical Instrumentation/ European Society of Hypertension/ International Organization for Standardization Collaboration
Last revision	1993	1999	2010	2013	2018
Sample size	85	96	33	≥85	≥85
Participants’ age	15–80 years	>20 years	≥25 years	>12 years	> 12 years
Age distribution	By chance	3 age groups Distribution based on age and SBP and DBP levels	By chance	By chance	By chance
Sex distribution	By chance	Equally represented	≥10 subjects of each sex	≥30% of each sex	≥30% of each sex
Arm circumference distribution	By chance	By chance	By chance	Single cuff: ≥40% in the upper/lower half of the specified cuff-range ≥20% in the upper/lower quarter. Multiple (n) cuffs: each cuff at least 1/(2 × n) of the subjects	Single cuff: ≥40% in the upper/lower half of the specified cuff-range ≥20% in the upper/lower quarter, ≥10% within the upper/lower octile Multiple (n) cuffs: each cuff at least 1/(2 × n) of the subjects
BP range distribution	SBP (mmHg) <90: ≥8 subjects, 90–129: ≥20, 130–160: ≥20, 161–180: ≥20, >180: ≥8, DBP (mmHg) <60: ≥8 subjects, 60–79: ≥20, 80–100: ≥20, 101–110: ≥20, >110: ≥8	20–40 years SBP (mmHg): ≤140:12 subjects ≥141:12 DBP (mmHg): ≤90:12 ≥91:12 41–70 years: SBP: ≤120:8 121–140:16 141–160:16 ≥ 161:8 DBP: ≤80:8 81–90:16 91–100:16 ≥101:8 ≥71 years: SBP: ≤140:12 >141:12 DBP: ≤90:12 >91:12	SBP (mmHg) <130:10–12 subjects, 130–160:10–12, >160 mmHg: 10–12 DBP (mmHg) <80:10–12, 80–100:10–12, >100:10–12	SBP (mmHg) ≤100 mmHg: ≥5% of readings ≥140 mmHg: ≥20% ≥160: ≥5%, DBP (mmHg) ≤60 mmHg: ≥5% ≥85 mmHg: ≥20% ≥100 mmHg: ≥5%	SBP (mmHg) ≤100 mmHg: ≥5% of readings ≥140 mmHg: ≥20% ≥160: ≥5%, DBP (mmHg) ≤60 mmHg: ≥5% ≥85 mmHg: ≥20% ≥100 mmHg: ≥5%
Reference BP measurement	Mercury sphygmomanometer	Mercury sphygmomanometer	Mercury sphygmomanometer	Mercury sphygmomanometers or non-mercury auscultatory device with maximum error ± 1 mmHg	Mercury sphygmomanometers or accurate non-mercury devices with maximum error ± 1 mmHg
Measurement method	Same arm sequential BP measurement	Same arm sequential BP measurement	Same arm sequential BP measurement	Same-arm sequential or simultaneous (same or opposite arm) BP measurement	Same arm sequential BP measurement
Paired BP measurements	255	≥288	99 (22–44 in each BP range)	255	255
Specific guidelines for ABPM	Yes	No	No	Yes (separate validation protocols)	Yes (separate validation protocols)
Pass criteria	Grading system (A, B, C, D) based on differences between paired readings by ≤ 5, 10, 15 mmHg separately for each observer and separately for SBP and DBP Additionally, mean differences ≤ 5 mmHg and SD ≤ 8 mmHg (AAMI recommendations)	Criteria based on mean difference and SD and point system for individual paired SBP and DBP readings Pass if the device fulfills all the following criteria: mean difference for SBP and DBP ≤ 5 mmHg and the SD ≤ 8 mmHg and point score ≥ 55% of the maximum attainable point score.	Criteria based on the number of readings with test-reference BP difference ≤ 5, 10, 15 mmHg Criteria for individual BP measurements (Part 1) individual subjects (Part 2) Part 1: Pass if 73.7% (73/99) of differences between readings: ≤ 5 mmHg, 87.9% (87/99) ≤ 10 mmHg, 97.6% (96/99) ≤ 15 mmHg Part 2 (Accuracy): number of subjects with 0, 2 or 3 of absolute difference ≤ 5 mmHg	Criteria based on mean BP differences and their SDs Criteria for individual BP readings and individual subjects. Criteria 1 and 2 should be applied for SBP and DBP Criterion 1 (for individual BP readings): The mean BP difference ≤ 5 mmHg and SD ≤ 8 mmHg Criterion 2 (for individual subjects): The mean difference and SD of BP readings within threshold defined by mean value of criterion 1	Criteria based on mean BP differences (test vs. reference) and their SDs Criteria for individual BP readings and individual subjects. Criteria 1 and 2 should be applied for SBP and DBP Criterion 1 (for individual BP readings): The mean BP difference ≤ 5 mmHg and SD ≤ 8 mmHg. Criterion 2 (for individual subjects): The mean difference and SD of averaged BP differences must be within a threshold defined by mean value of criterion 1 Additionally, the number of absolute BP differences within 5, 10, and 15 mmHg (ESH-IP2) and standardized Bland–Altman scatterplots will be presented The mean test-reference BP difference and SD per cuff subgroup must be reported without pass/fail criteria for the test device
Special groups	Pregnant women, elderly, and children Only if a device has successfully completed all phases of Part I and has achieved at least a B grading for accuracy for both SBP, DBP (Part II.I)	Pregnant women, diabetics, arm circumference > 33 cm	No specific guidelines Separate studies recommended for special populations	Pregnant women, neonates and children, heart irregularities/disease	Children < 3 years, pregnancy including preeclampsia, arm circumference > 42 cm, atrial fibrillation Possible special groups: individuals aged 12–21 or >80 years and those with end-stage kidney disease Special population studies with smaller sample sizes should be performed only after a full general population study has been successfully completed. If the device is intended only for a special population, then a full 85-subject study is required
Special occasions	During exercise and in various postures. Only if a device has successfully completed all phases of Part I and has achieved at least a B grading for accuracy for both SBP, DBP (Part II.II)	No specific guidelines	No specific guidelines	During exercise	No specific guidelines
Extra	Examines intradevice variability, accuracy of devices after long term period of performance				

BP, blood pressure; SBP, systolic blood pressure; DBP, diastolic blood pressure; ABPM, ambulatory blood pressure monitoring; SD, standard deviation.

### Sample size

A major difference between protocols is that the ESH-IP2 is the only one that requires a smaller sample size of 33 individuals instead of 85 and 96 individuals that the other protocols require.

### Age of participants

Most of the protocols are designed for adults, whereas the ANSI/AAMI/ISO and the AAMI/ESH/ISO include children older than 12 years.

### Distribution of participants

In all protocols, the inclusion criteria for the participants vary in regard to their age, sex, arm circumference, and entry BP distribution. The sample is distributed based on age only in DHL, whereas in the others the distribution is random. Most of the protocols, except from the BHS, include participants selected with sex criteria. Only the ANSI/AAMI/ISO and AAMI/ESH/ISO protocols use sample that is selected according to the arm circumference. Finally, all protocols use BP range as a criterion for the selection of the subjects.

### Measurement method

All protocols endorse the same-arm sequential measurement as the most accurate method except for the ANSI/AAMI/ISO, which suggest alternatively the same or the opposite arm simultaneous BP measurement procedure.

### Reference blood pressure measuring device

In the BHS, DHL, and ESH protocols, the recommended reference BP measurement device is a mercury sphygmomanometer, whereas the protocols of ANSI/AAMI/ISO and AAMI/ESH/ISO suggest alternatively the use of other non-mercury devices with a maximum error of 1 mmHg.

### Pass/Fail criteria

Different pass criteria have been used in all the protocols. The DHL, AANSI/AAMI/ISO, and AAMI/ESH/ISO criteria are based on calculating the mean difference and the standard deviation (SD) between the test and the reference BP measuring devices. The DHL has used additionally a point system scoring. On the other hand, the BHS and the ESH-IP2 criteria are based on summing up the cumulative incidence of the difference between the test and the reference BP devices in the categories of less than 5, 10, and 15 mmHg.

### Specific guidelines for ABPM

Only the BHS, ANSI/AAMI/ISO and the AAMI/ESH/ISO protocols highlight the need of separate validation studies for ABPM.

### Special occasions (such as exercise)

The BHS includes specific guidelines for the validation of BP devices in special occasions, such as during exercise and in different postures, whereas the AAMI provides recommendations for the validation of BP monitors only during exercise.

### Special populations (such as pregnant women, children, elderly, and patients with diabetes)

All the protocols recognize that BP devices should be validated in special populations and provided specific recommendations for these groups except for ESH-IP2 that recommends separate studies to be carried out.

Finally, the BHS protocol is the only one that tests the intradevice variability and the consistency in the performance of the BP monitor after prolonged use.

## Validation protocols in children and adolescents

The recommendations for validation of BP measurement devices are mainly “tailor made” for adults. Although some organizations have addressed the validation in special populations including children, they mostly consider children as “small adults” and do not take into account several distinct characteristics of the pediatric population. Finally, most of the documents on the validation of BP measuring devices have included in the writing committees only adult hypertension specialists putting less emphasis on this special population. Given that the scientific evidence beyond the recommendations is limited and all organizations provide consensus documents the lack of statements on the validation of BP measuring devices specifically addressing to the unique characteristics and needs of children and adolescents by specialists and practitioners caring exclusively for pediatric patients gains extreme importance as a fundamental step for accurate and reliable BP measuring devices in the pediatric population.

The BHS, ANSI/AMI/ISO, and AAMI/ESH/ISO are the only protocols, which include specific recommendations for the validation of BP measuring devices in children (3, 9, 10) (Table 2). According to BHS, a sample of 30 children aged 5–15 years with specific inclusion criteria for their age, sex, and entry BP distribution is required (3). Afterwards, the mean BP difference and SD between test and reference device measurements should be reported without specified pass criteria. The ANSI/AMI/ISO and AAMI/ESH/ISO protocols share the same principles (9, 10). If the device is intended for use on both adults and children, the sample should consist of 35 children aged 3–12 years and 50 individuals aged older than 12 years. On the other hand, if the device is intended only for the use on children, a study with a sample of 85 children with specific criteria for sex and cuff size distribution should be carried out. According to the protocols, the studies should meet both two criteria for BP differences of individual readings and of individual subjects. The criterion 1 defines that the mean BP difference (test minus reference BP for all of the measurements) must be 5 mmHg or less, and its SD 8 mmHg or less for systolic and diastolic BP and the criterion 2 that the SD of averaged BP differences (test minus reference BP per subject) must be within a threshold defined by the mean of criterion 1 (9).

**TABLE 2:** Comparison of validation protocols for BP measuring devices in children.

	BHS revised (1993) (3)		ANSI/AAMI/ISO (2013) (7)		AAMI/ESH/ISO (2018) (8)		
	After a successful study in general population		Device for children and adults or with pediatric mode:	Devices only for children:	Devices for both general population and children: (after a successful 85-subject study in general population)	Devices with a special pediatric mode: (after a successful 85-subject study in general population)	Devices only for children: (without previous study in general population)
Age range	0–5 years	5–15 years	3–12 years		3–12 years and ≥ 12 years	3–12 years	3–12 years
Sample	30 subjects	30 subjects	35 subjects	85 subjects	85 subjects	35 subjects	85 subjects
Age distribution	0–12 months: 15 subjects, 1–5 years: 15	Evenly distributed	Not specified		3–12 years: 35 subjects, > 12 years: 50	Not specified	Not specified
Sex distribution	≥10 each of sex	By chance	≥30% of each sex		≥30% of each sex		
BP range distribution	**SBP:** 5/30 > mean + 1 SD for population **DBP:** 5/30 < mean—1 SD for population	**SBP:** 5/30 > mean + 1 SD for population 5/30 < mean—1 SD for population **DBP:** 4/30 > mean + 1 SD for population 5/30 < mean—1 SD for population	Not specified		As the total 85-subject study	Without BP distribution requirements	
Arm circumference distribution	Not specified	5/30 > 70th centile for weight 5/30 < 30th centile for weight	Single cuff: 40% of subjects’ circumference within upper half of range; 40% within lower half; 20% of subjects’ circumference within upper quarter of range; 20% within lower quarter. N cuffs, test each in ≥ 1/(2 × n) subjects		Single cuff: 40% of subjects’ circumference within upper half of range; 40% within lower half; 20% of subjects’ circumference within upper quarter of range; 20% within lower quarter. N cuffs, test each in ≥ 1/(2 × n) subjects		
Reference BP measurement device	Conventional mercury sphygmomanometry	Mercury sphygmomanometer, or non-mercury auscultatory device with max permissible error ± 1 mmHg	Mercury sphygmomanometer, or non-mercury auscultatory device with max permissible error ± 1 mmHg		
Reference diastolic BP	K5	K4	K4		
Pass criteria	Mean difference and SD for test-reference BP differences to be reported No pass threshold is provided	Criterion 1: mean ± SD for test-reference BP differences ≤ 5 ± 8 mmHg Criterion 2: intersubject SD of BP differences within threshold defined by the mean of criterion 1	Mean difference and SD of SBP and DBP should be reported separately for subgroups aged 3–12 and > 12 years Pass criteria: validation criteria 1 and 2 Criterion 1: mean ± SD for test-reference BP differences ≤ 5 ± 8 mmHg Criterion 2: intersubject SD of BP differences within threshold defined by the mean of criterion 1	Validation criterion 1	Validation criteria 1 and 2

BP: blood pressure, SBP: systolic blood pressure, DBP: diastolic blood pressure, SD: standard deviation.

### Considerations on validation protocols in children and adolescents

#### Population and sample size

The optimal sample size for a BP measuring device validation study varies among different organizations. As mentioned above the ESH validation protocol suggested a minimum of 33 subjects, while the BHS, the ANSI/AAMI/ISO as well as the AAMI/ESH/ISO required 85 participants (3, 10). The disagreement on ideal population sample sizes lies on the statistical power of the validation procedure against the cost and complexity (10, 12).

In the AAMI/ESH/ISO consensus statement, it was reported that that a validation study with a sample size of 35 subjects would be inadequate for a moderate accuracy device defined as a difference of 4 ± 5 mmHg compared to the test device, because of an unacceptably high at 28% chance to fail (10). However, according to a biostatistician report, a validation study with 35 subjects would be adequate for high- or low-accuracy devices. Then, it was calculated that a population sample of 85 subjects as previously suggested by the ANSI/AAMI/ISO has an acceptable chance of failing (18%) supporting the previous consensus of at least 85 subjects and taking into account that most devices in the market probably have moderate accuracy.

Adolescents older than 12 years are considered as general population and are evaluated within an 85-population sample. Transfer functions and in-built algorithms for the calculation of systolic and diastolic BP are not usually available by the manufacturer (13). The algorithms differ between devices, are considered proprietary for the manufacturer, and are, therefore, confidential. Of note, these algorithms are developed for adults with higher BP levels, and automated initial cuff inflations to high pressures may cause discomfort or pain in the child precluding its cooperation (14). Oscillation may also be lower in the youngest with lower BPs. For example, for a 12-year-old-boy with short stature at the 5th centile, the median level (50th centile) of systolic and diastolic BP is at 101/65 mmHg, respectively. Then, it is well described that in adolescents, the pulse wave contour is different than in older individuals with stiffer arteries (15). Whether these algorithms could evaluate with the same accuracy, the BP level in an adolescent as young as 12 years old and in a 65-year-old individual remains unanswered and uninvestigated.

A low-accuracy device for adolescents with an in-built algorithm resulting in high accuracy in older subjects would result in a medium-accuracy device with the inclusion of subjects 12–18 years in general population study. While the impact of this result would be moderate for the adult population, it may have important implications for adolescents regarding misclassification of their BP status and possibly undiagnosed hypertension.

The ESH 2016 guidelines on the management of high BP in children and adolescents consider that only older adolescents (≥16 years) are evaluated for hypertension using the adult BP threshold (16). It may be prudent that this age limit of 16 years would also apply for the validation studies in the general population. Then, a separate validation study considering adolescents <16 years as a special population may offer the opportunity for more precise assessment of accuracy before a device is suggested in the adolescent age range.

Children are considered as a special population if younger than 12 years. According to the BHS, the number of pediatric patients 3–12 years needed for a BP measurement device validation study is 30, if the device has been successfully validated in the general population. The ANSI/AAMI/ISO and the AAMI/ESH/ISO recommend a sample size is of at least 85 subjects if the study includes only children, but in the case of an existent validation study for the general population, the required sample size is 35 children. For validation, studies including both children and adults’ general population, a total sample size of at least 85 is required, with children consisting of 35 out of 85 participants. The same concerns about the transfer functions and in-built algorithms may apply for children 3–12 years. Again, given all the above considerations, it is unclear if the sample of 35 children would be adequate for this age range with low oscillation and different vascular functions for moderate accuracy devices (13).

#### Cuff size

Most monitors included two cuffs for the adult population. Special-size cuffs are not always available and in case of minors, children, and adolescents, this is an important issue. In the same concept as in the previous section, younger adolescents may erroneously be considered as general populations as they have different characteristics. It is recommended that the cuffs used for reference auscultatory BP measurement must have an inflatable bladder length that covers 75–100% of the upper arm circumference of each participant and a width that covers 37–50% of the arm circumference measured at the upper arm midpoint between acromion and olecranon (10). Many manufacturers include adult cuffs that are suitable for arm length >22 cm. For 12–15–years-old girls, the 5th–25th centile of midarm circumference is <20 cm and adult cuffs are not suitable for reliable BP measurement (17, 18). Similarly, the 25th centile of midarm circumference of a 12-year-old boy is <20 cm and for 14- and 15-years-old boys is at 22 and 23 cm, respectively. In the AAMI/ESH/ISO, it is recommended that inflatable bladder dimensions should be 12 cm for 12–15 years old and 15 cm for 15–18 years old.

If a device is considered for validation in children and adolescents, commercially available cuffs sizes both for the validation study but also for routine use should be a prerequisite criterion. Although not specifically reported in the consensus documents using cuffs from other manufacturers or from the test device, not designed for the device under evaluation, for the reference BP measurement during the validation study may result in significant measurement errors and significant bias of the validation study methodology.

#### Diastolic blood pressure

The latest ESH and American Academy Pediatrics (AAP) guidelines for the diagnosis of high BP in children and adolescents recommend the use of Korotkoff sound 5 (K5) during office BP measurement (16, 19). The most frequently used validation protocol, the ANSI/AAMI/ISO recommends the use of Korotkoff sound 4 (K4) during the validation procedure which constitutes a major inconsistency between validation and clinical use of a device (9). However, in line with the guidelines for diagnosis of the hypertension in children and adolescents, the BHS, as well as the universal AAMI/ESH/ISO 2018 protocol recommend the use of K5 (3, 20). The latter recommends that if K5 is not audible, the child should be excluded.

#### Validation criteria

In all consensus validation documents, two criteria as defined by the ANSI/AAMI/ISO are used to evaluate the successful validation of devices usually reported as pass or fail in review articles (9). The same criteria apply for pediatric studies although no studies have been performed to evaluate the suitability of these criteria in pediatric patients. However, only criterion 1 is necessary to be reported in the case of 35 subject studies. Of note, in case of a validation study including children in a general population study, both criteria should be reported separately for the pediatric subgroup.

## The example of validated office blood pressure devices in children and adolescents

A systematic search using Medline from inception to May 30, 2022, was performed to identify studies validating the accuracy of office BP monitors in the pediatric population alone or as a subgroup of the study population. We used the following search terms: (Office) AND (Blood Pressure) AND (Validation) AND (Monitor) OR (Device) AND (Children) OR (Adolescents). A hand-searching was also conducted for eligible studies. The reference list of each article included was checked for extra bibliography. Duplicates were removed. We included studies in the English language only. Two independent reviewers (KE and CS) screened titles and abstracts independently, and full texts were investigated for eligible studies. Differences between the two reviewers regarding study eligibility were resolved by a third reviewer (SS). Finally, study and population characteristics were extracted from each included study.

**FIGURE 1:**
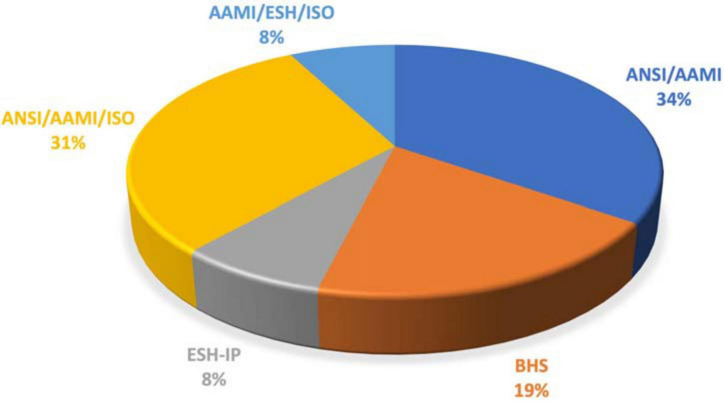
Validation protocols used in studies assessing accuracy of BP measuring devices in children and adolescents.

The search resulted in 21 studies with successful validation in children and adolescents (Supplementary Figure 1) (21–39). Validated devices for office BP measurement, children and adolescents using different available validation protocols are presented in Table 3. The accuracy of BP measuring devices was assessed using the ANSI/AAMI and the AMSI/AAMI/ISO protocol in almost 80% of the validation studies in children and adolescents (Figure 1). About half of the studies were performed before 2010. Few office BP devices were validated based on two different protocols, both the ANSI/AAMI and the BHS protocols (*n* = 4) (21, 29, 37, 38) or the BHS and the ESH-IP (*n* = 2) (23, 24, 39). In all studies, devices passed the validation criteria by both protocols for systolic and diastolic BP with the exception of the Dinamap Procare-200 device that failed for the diastolic BP with ESH protocol criteria (24). One device that has been assessed by two studies was evaluated as passed in one of them but failed in the other one (23, 39). In one study, 3 devices were evaluated simultaneously (23).

**TABLE 3:** Validated devices for office BP measurement in children and adolescents.

Device (References)	No. of patients/ age range	No. of pediatric patients/ age range	Validation protocol	Result	Test device	DBP definition (K4 or K5)	Cuff sizes used	Device commercially available cuff sizes	Funding
**Successful validation including only children**									
BpTru BPM-100 (BpTRU Medical Devices, Canada, USA) (21)	36/3–18 years	36/36 (3–18 years)	ANSI/AAMI, BHS	Pass, grade A	Auscultatory mercury sphygmomanometer	K5	na	Child 13–18 cm, Small adult 18–26 cm, Regular adult 26–34 cm, Large adult 32–43 cm, Extra−large adult 41–52 cm	nr
CasMed 740 (CAS Medical Systems, Branford, Connecticut, USA) (22)	29/ < 3 years	29/29 (29 days–1 year: 5, 1–3 years: 3)	ANSI/ AAMI/ISO	Pass	Invasive arterial measurement	na	Neonate cuffs: #1: 3–6 cm #2: 4–8 cm #5: 8–15 cm, Child cuff: 13–20 cm	Neonate: #1: 3–6 cm #2: 4–8 cm #3: 6–11 cm #4: 7–14 cm #5: 8–15 cm, Infant 8–14 cm, Child 13–20 cm, Small adult 18–26 cm, Adult 26–35 cm, Adult long 29–38 cm, Large adult 32–42 cm, Large adult long 35–44 cm, Adult thigh 42–50 cm,	CAS Medical Systems Inc.
Datascope Accutorr Plus (Datascope Corporation, Mahwah, New Jersey, USA) (23)	44/5–15 years	44/44 (5–15 years)	ESH	Pass	Auscultatory mercury sphygmomanometer	K5	na	9–14.8 cm orange, 13.8–21.5 cm green, 20.5–28.5 cm light blue, 27.5–36.5 cm navy blue, 35.5–46 cm burgundy, 45–56.5 cm brown	Hong Kong Paediatric Nephrology Society, Children’s Kidney Trust Fund
Dinamap Procare-120 (Critikon, Tampa, Florida, USA) (23)	44/5–15 years	44/44 (5–15 years)	ESH	pass/fail	Auscultatory mercury sphygmomanometer	K5	na	Infant, Child, Small adult, Adult, Large adult, Adult thigh	Hong Kong Paediatric Nephrology Society, Children’s Kidney Trust Fund
Dinamap Procare-200 (Critikon, Tampa, Florida, USA) (24)	45/(7–18 years)	45/45 (7–18 years)	BHS, ESH	pass, pass/fail	Baumanometer Mercury Gravity Sphygmomanometer (W.A. Baum Co., Copiague, NY, USA)	na	Child 17–22 cm, Small adult 22–30 cm, Large adult 30–38 cm	Neonate Neo #1 Neo #2 Neo #3 Neo #4 Neo #5, Infant, Child, Small adult, Adult, Large adult, Adult thigh, Adult long cuff *(Different assortment packs available)*	Korea Center for Disease and Prevention (KCDC)
**Successful validation including only children**									
Raycome RBP-1200 (Shenzhen Raycome Health Technology, China) (25)	3–12 years	87/87 (3–12 years)	ANSI/AAMI/ISO	Pass	Auscultatory mercury sphygmomanometer	K5	Extra small 15–18 cm, small 18–22 cm, standard 23–32 cm	Extra small (SS) 15–18 cm, Small (S) 18–22 cm	National Nature Science Foundation of China, Shenzhen Raycom Health Technology Company (Shenzhen, China)
**Successful validation both adults and children**									
CAS 9010 (CAS Medical Systems, Branford, Connecticut, USA) (27)	4–78 years	35/88	AAMI	Pass	Auscultatory mercury sphygmomanometer	na	na	na	nr
Colin BP8800MS (Colin Medical Instruments Corp., San Antonio, TX) (28)	170, 8 months–80 years	85/170 (8 months–16 years)	ANSI/AAMI	Pass	Mercury manometer (model Marshall Deluxe, Omron Healthcare, Inc., Vermon Hills, IL)	K5	Infant 8–12.5 cm, Child 12.5–18 cm, Small adult 18–24 cm, Adult 24–32 cm, Large adult 32–40 cm	Neonate #1 #5, Infant, Child, Small adult, Adult, Large adult	Nippon Colin (Komaki, Japan) and Colin Medical Instruments (San Antonio, TX)
Fukuda Denshi DS-7000/NIBP-701 (Fukuda Denshi Co., Tokyo, Japan) (29)	119/42.2 ± 21.0 years	33/119 (15 pediatric (3–12), 18 neonate/infant (< 3))	ANSI/AAMI, BHS	Pass, pass-grade A	Intraarterial-neonates and infants, auscultatory-children and adults	na	na	Infant cuff, Child cuff, Large/Regular/Small Adult cuff	Fukuda Denshi
Microlife WatchBP Office (Microlife AG, Widnau, Switzerland) (30)	88/3–70 years	37/88 (3–12 years)	ANSI/AAMI/ISO	Pass	Auscultatory mercury sphygmomanometer (Baumanometer; WA Baum Co., Inc., New York, New York, USA)	K5	Small 14–22 cm, Medium 22–32 cm, Large 32–42 cm	Medium 22–32 cm, Large 32–42 cm	Microlife, Widnau Switzerland, University of Athens Special Account for Research Grants
Midmark IQvitals Zone (Midmark Corporation, USA) (31)	85/3–77 years	35/85 (7–17 years)	ANSI/AAMI/ISO	Pass	Manual auscultation	K5	na	Child, Small adult, Adult, Adult long, Large adult, Large adult long, Thigh	Midmark Corporation
Nihon Kohden PVM-2701/Impluse-1 (32)	110/na	41/110 (<12 years)	ANSI/AAMI/ISO	Pass	Auscultatory mercury sphygmomanometer	K4	na	Infants 8–13 cm, Children: Small 12–18 cm Standard 15–23 cm, Adults: Standard 21–30 cm Large 23–36 cm, Thigh 33–45 cm	University of Tennessee Clinical Research Center and the Tennessee Clinical Trials Network, Nihon Kohden
Omron HBP-1300 (Omron Healthcare Co., Kyoto, Japan) (33)	85/4–72 years	35/85 (<12 years)	ANSI/AAMI/ISO	Pass	Auscultatory mercury sphygmomanometer	K4	SS 12–18 cm, S 17–22 cm, M 22–32 cm, L 32–42 cm, XL 42–50 cm	SS 12–18 cm, S 17–22 cm, M 22–32 cm, L 32–42 cm, XL 42–50 cm,	Guangzhou Boji Medical Biotechnological Co. Ltd.
**Successful validation including only children**									
Omron HBP-1320 (Omron Healthcare Co., Kyoto, Japan) (34)	88/4–70 years	38/88 (4–12 years)	ANSI/AAMI/ISO	pass, pass	Auscultatory mercury sphygmomanometer	K5	SS 12 to 18 cm, S 17 to 22 cm, M 22 to 32 cm, L 32 to 42 cm, XL 42 to 50 cm	SS 12–18 cm, S 17–22 cm, M 22–32 cm, L 32–42 cm, XL 42–50 cm	nr
Omron M3500 (Omron Healthcare Co., Kyoto, Japan) (35)	135./≥3 years	35/135 (3–12 years)	ANSI/AAMI/ISO	Pass	auscultatory mercury sphygmomanometer	K4 and K5	Super small: 12–18 cm, Small: 17–22 cm, Standard: 22–32 cm, Large: 32–42 cm, Xlarge: > 42 cm	*Standard* IEC adult cuff or GS cuff M *Optional* IEC adult oversized cuff IEC children cuff (9 cm, 7 cm), Adult cuff (S, M, L, XL), GS cuff (SS, S, L, XL)	OMRON (Japan)
Welch Allyn Pro BP 2000 (Welch Allyn, Skaneateles Falls, New York, USA) (36)	88/ ≥3 years	35/88 (3–12 years)	ANSI/AAMI/ISO	Pass	Auscultatory mercury sphygmomanometer	K4	Child 15–21 cm, Small adult 20–26 cm, Adult 25–34 cm, Adult long 32–43 cm, Adult large long 32–43 cm, Thigh 40–55 cm	Child 15–21 cm, Small adult 20–26 cm, Adult 25–34 cm, Adult long 32–43 cm, Adult large long 32–43 cm, Thigh 40–55 cm	Welch Allyn
Welch Allyn ProBP 3400 (Welch-Allyn Medical Products, New York, USA) (37)	111, ≥3 years	14/111 (3–12 years)	ANSI/AAMI, BHS	Pass, pass-grade A	Auscultatory mercury sphygmomanometer	K5	na	Small child (12 cm) to thigh (55 cm)	nr
Welch Allyn SureBP, StepBP (Welch-Allyn Medical Products, New York, USA) (38)	102/ ≥3 years	15/102 (3–12 years)	ANSI/AAMI, BHS	Pass, grade A	Auscultatory mercury sphygmomanometer	K5	na	Extra small 15–24 cm, Standard wide 22–42 cm, Extra large 40–54 cm	nr
Welch-Allyn Spot Vital Signs (Welch-Allyn Medical Products, New York, USA) (39)	5–77 years, 47 ± 16	na, >5 years	ANSI/AAMI	Pass	Auscultatory mercury sphygmomanometer (Tycos brand sphygmomanometer; Tycos, Inc., Arden, North Carolina, USA)	K5	na	Neonate: #1: 3.3–5.6 cm #2: 4.2–7.1 cm #3: 5.4–9.1 cm #4: 6.9–11.7 cm #5: 8.9–15 cm, Infant 9.8–13.3 cm, Small child 12.4–16.8 cm, Child 15.8–21.3 cm, Small adult 20–27 cm, Adult 25.3–34.4 cm, Large adult 32.1–43.4 cm, Thigh 40.7–55 cm	Welch Allyn, Inc.
YuWell YE900 (Jiangsu Yuyue Medical Equipment and Supply, China) (26)	85/3–12 years	35/85 (4–11 years)	AAMI/ESH/ISO	Pass	Auscultatory mercury sphygmomanometer (YuYue, Jiangsu Province, China)	K5	18–22 cm (small), 22–32 cm (medium), and 32–42 cm (large)	na	YuYue Medical Equipment & Supply Co., Ltd.

DBP, diastolic blood pressure; na, not available; nr, not reported.

Only 6 out of 7 studies that included exclusively pediatric population fulfilled the pass criteria. Three used the ESH-IP protocol (23, 24), which is not designed for children, two the BHS protocol, and three the ANSI/AAMI/ISO protocol. Among studies that used the ANSI/AAMI/ISO protocol, two had an inadequate sample size, leaving only 1 study that used the ANSI/AAMI/ISO protocol to provide the best available validation evidence for office BP devices in children (25).

The test device used in almost all studies was a mercury sphygmomanometer measuring BP by the auscultatory method. Intra-arterial measurement as a test method was used in 2 studies (22, 29), one of them including only neonates and infants (29). Most studies included two trained observers for the BP measurements as recommended and most of them were health professionals.

Seven out of twenty-one validating studies did not meet the criteria for the age range required based on the selected protocols. None of the studies reported the required age distribution in the population. Only five studies met both the sample age and sample size required for a validation study. Although the available protocols do not specify the required ratio of female–male participants for children’s studies, most of the studies that defined their population, recruited the same percentage of patients of each sex.

Among studies that used the AAMI protocol the one that included 85 children reported both validation criteria 1 and 2. Also in studies including both the general population and children both validation criteria 1 and 2 were used. In the studies including 35 children with an existent validation study for adults, only criterion 1 was used. Funding by the industry was reported in eight studies (25, 28–33, 35, 36).

### Pitfalls during validation procedure

Several validation studies in children or including children in the general population lack adequate reporting of validation data according to validation recommendations or not fulfill all requirements (Table 3). Common pitfalls include:

#### Sample size

The requirements for sample size were satisfied in 12 out of 21 studies. For example, the validation study by Alpert et al., using the ANSI/AAMI/ISO protocol included only children, but the sample size was less than the required sample size of at least 85 subjects (36). The same number of subjects was included by Mattu et al. (21) in a validation study for a BP measuring device intended for use in children but considered adequate as there was already an existent validation study for the general population (21).

#### Cuff size

In about half of the studies, no data were reported regarding the cuffs used for the validation procedure. Moreover, in several cases, information about commercially available cuffs for the validated device was not reported in manufacturer’s sites. Manufacturers may provide only one adult cuffs with the device and pediatric cuff sizes are usually sold separately as extra accessories. In the validation studies that included both children and adults, 6 out of 13 studies used for the validation procedure the cuffs provided by the manufacturer along with the device (Table 3). Cuff sizes used during the validation with the description of cuffs’ length and width were usually reported, but only 4 of them reported the number of subjects tested for different cuff sizes. In the validation studies that included only children, 2 out of 6 studies used the same size cuffs as provided for the validation, and only 3 out of 6 studies reported the number of subjects tested for each cuff size. Details on the selection of cuff size, midarm circumference of the population and/or midarm circumference by cuff size used were scarcely reported.

#### Definition of diastolic blood pressure

Most of the included studies used K5 for the definition of diastolic BP, as it is suggested by BHS and AAMI protocols. Some studies didn’t report by which Korotkoff sound (K4 or K5) was diastolic BP defined. K4 was reported in four studies while one study reported both K4 and K5 for all participants (35). Five studies used ANSI/AAMI/ISO protocol but reported K5 (25, 26, 30, 31, 33).

#### Validation criteria

Validation criterion 1 was used in all studies. Results for children were reported together with those of older participants (adults) in the case of studies in the general population, and only one study (1 out of 13) reported data on criterion 1 separately for children (28).

## Conclusion

The level of evidence-based upon pediatrics studies for the established validation criteria in children and adolescents needs to be assessed to evaluate the suitability of these criteria in children and adolescents. When evidence does not exist then extrapolation of data from adult studies is usually applied, but limitations of such strategy need to be acknowledged and gaps of evidence would serve as motivation for designing the new research activities. This is the case for BP measuring devices validation studies in children and adolescents. In addition, most validation studies analyzed children’s data along with adult ones posing significant uncertainty on the accuracy of the BP measuring validated devices in the pediatric population. Given that automated oscillometric BP devices become extensively popular in routine clinical practice for the diagnosis of high BP in childhood the need of validation protocols addressing the needs and special characteristics of children and adolescents is emerging to ensure accurate evaluation of BP levels in childhood.

## Author’s note

This publication is based on the work of the COST Action HyperChildNET (CA19115).

## Author contributions

SS an EL: conceptualization. SS, AC, KE, CS, CA, PP, JC, and GH: writing—original draft preparation. SS and AC: writing—review and editing. All authors have read and agreed to the current version of the manuscript.
