# Supplementary material for: Comparison of validation protocols for blood pressure measuring devices in children and adolescents
Source: Front Cardiovasc Med. 2022 Nov 23;9:1001878. doi: 10.3389/fcvm.2022.1001878 (PMC9727228; doi:10.3389/fcvm.2022.1001878)
Supplement: Supplementary file 1 [file Data_Sheet_1.docx]

**Supplementary Figure 1.** Flow Chart for study selection process

Studies included
(n = 21)

## Identification

## Included

## Eligibility

## Screening

Records identified through database searching
(n = 601)

Additional records identified through other sources (hand searching)
(n = 36)

Full-text articles excluded

(Unsuccessful validation)
(n = 4)

Records excluded
(n = 12)

Full-text articles assessed for eligibility
(n = 25)

Records screened
(n = 37)

Records after duplicates removed
(n = 634)
